# Supplementary material for: Comparative genomics provides new insights into the diversity, physiology, and sexuality of the only industrially exploited tremellomycete: Phaffia rhodozyma
Source: BMC Genomics. 2016 Nov 9;17:901. doi: 10.1186/s12864-016-3244-7 (PMC5103461; doi:10.1186/s12864-016-3244-7)
Supplement: Additional file 6: — List of orphan genes with links to PFAM (related to Additional file 1: Table S1). (ZIP 1428 kb) [file 12864_2016_3244_MOESM6_ESM.zip › BLAST_HTML_FTR/G00023_P.html]

BLAST Search Results


```
BLASTP 2.2.27+


Reference:
Stephen F. Altschul, Thomas L. Madden, Alejandro A. Schäffer,
Jinghui Zhang, Zheng Zhang, Webb Miller, and David J. Lipman (1997),
"Gapped BLAST and PSI-BLAST: a new generation of protein database
search programs", Nucleic Acids Res. 25:3389-3402.


Reference for
composition-based statistics:
Alejandro A. Schäffer, L. Aravind, Thomas L. Madden, Sergei
Shavirin, John L. Spouge, Yuri I. Wolf, Eugene V. Koonin, and
Stephen F. Altschul (2001), "Improving the accuracy of PSI-BLAST
protein database searches with composition-based statistics and
other refinements", Nucleic Acids Res. 29:2994-3005.


Database: nr
           71,551,133 sequences; 26,053,659,533 total letters


Query= G00023_P

Length=1031
                                                                      Score     E
Sequences producing significant alignments:                          (Bits)  Value

emb|CED84332.1|  hypothetical protein [Xanthophyllomyces dendrorh...  2069    0.0  


 >emb|CED84332.1| hypothetical protein [Xanthophyllomyces dendrorhous]
Length=1030

 Score = 2069 bits (5361),  Expect = 0.0, Method: Compositional matrix adjust.
 Identities = 1030/1030 (100%), Positives = 1030/1030 (100%), Gaps = 0/1030 (0%)

Query  1     MARTNIWQGSLRTPSKPLDGICITSLSSGESVALPSPPPTSSSSTYLDAQTSLITSRSSI  60
             MARTNIWQGSLRTPSKPLDGICITSLSSGESVALPSPPPTSSSSTYLDAQTSLITSRSSI
Sbjct  1     MARTNIWQGSLRTPSKPLDGICITSLSSGESVALPSPPPTSSSSTYLDAQTSLITSRSSI  60

Query  61    QFKHHAHPSSAETSSPPVQRTQIRKIKLNLTAGRPAAPKVAAIKSWIDRLKNEASEGEFV  120
             QFKHHAHPSSAETSSPPVQRTQIRKIKLNLTAGRPAAPKVAAIKSWIDRLKNEASEGEFV
Sbjct  61    QFKHHAHPSSAETSSPPVQRTQIRKIKLNLTAGRPAAPKVAAIKSWIDRLKNEASEGEFV  120

Query  121   VREGPDSVVKLADLDSGVLIHSVEEDLRSGYKIRATHSTQPSGLLYFHAALIPEPKAASL  180
             VREGPDSVVKLADLDSGVLIHSVEEDLRSGYKIRATHSTQPSGLLYFHAALIPEPKAASL
Sbjct  121   VREGPDSVVKLADLDSGVLIHSVEEDLRSGYKIRATHSTQPSGLLYFHAALIPEPKAASL  180

Query  181   SLDTQGRTLHGLLIDFVPFDPDHDVPHLTLLIYGIKSTPLALNSETERKKSKKPSLSIFA  240
             SLDTQGRTLHGLLIDFVPFDPDHDVPHLTLLIYGIKSTPLALNSETERKKSKKPSLSIFA
Sbjct  181   SLDTQGRTLHGLLIDFVPFDPDHDVPHLTLLIYGIKSTPLALNSETERKKSKKPSLSIFA  240

Query  241   VPFPVSTTPLSPPPSAASIESAISSSAPSTPCLLSASYSQMSAPGSTPSCRLRPRGTFSP  300
             VPFPVSTTPLSPPPSAASIESAISSSAPSTPCLLSASYSQMSAPGSTPSCRLRPRGTFSP
Sbjct  241   VPFPVSTTPLSPPPSAASIESAISSSAPSTPCLLSASYSQMSAPGSTPSCRLRPRGTFSP  300

Query  301   RHPRLSNVAIASSILSSSREPSPIPIVSPAQLPRPSKTEAESTDVPVVATSKTVSQPTPS  360
             RHPRLSNVAIASSILSSSREPSPIPIVSPAQLPRPSKTEAESTDVPVVATSKTVSQPTPS
Sbjct  301   RHPRLSNVAIASSILSSSREPSPIPIVSPAQLPRPSKTEAESTDVPVVATSKTVSQPTPS  360

Query  361   TSVPSSTTSPTQSCYTSSLDGQKTVNIAKNTSFPTSKFTFTAPSAIPAKVSSDRSVAPST  420
             TSVPSSTTSPTQSCYTSSLDGQKTVNIAKNTSFPTSKFTFTAPSAIPAKVSSDRSVAPST
Sbjct  361   TSVPSSTTSPTQSCYTSSLDGQKTVNIAKNTSFPTSKFTFTAPSAIPAKVSSDRSVAPST  420

Query  421   TIKRIVLKKSSLYLTSKPNIETAQAQGDIEQKNILPSSDSSESSSSSPSSDSDSDADEEK  480
             TIKRIVLKKSSLYLTSKPNIETAQAQGDIEQKNILPSSDSSESSSSSPSSDSDSDADEEK
Sbjct  421   TIKRIVLKKSSLYLTSKPNIETAQAQGDIEQKNILPSSDSSESSSSSPSSDSDSDADEEK  480

Query  481   KGGSQEENTSARKSREERDLKAERVEEKGRRERLVGNNQGKSTDWIHQNTQSVQSDLSPL  540
             KGGSQEENTSARKSREERDLKAERVEEKGRRERLVGNNQGKSTDWIHQNTQSVQSDLSPL
Sbjct  481   KGGSQEENTSARKSREERDLKAERVEEKGRRERLVGNNQGKSTDWIHQNTQSVQSDLSPL  540

Query  541   KYSSKSPSTVAPPLPRRSSNSKTQVELSRHILPLRKPTSITTNTRPAYVNTRPNIRTQLK  600
             KYSSKSPSTVAPPLPRRSSNSKTQVELSRHILPLRKPTSITTNTRPAYVNTRPNIRTQLK
Sbjct  541   KYSSKSPSTVAPPLPRRSSNSKTQVELSRHILPLRKPTSITTNTRPAYVNTRPNIRTQLK  600

Query  601   TQSTRPPPPPDVGFSHPFSSDGEVGEGPIKPIKSELFKDKVSRGDVLIEQDSEDVITSPA  660
             TQSTRPPPPPDVGFSHPFSSDGEVGEGPIKPIKSELFKDKVSRGDVLIEQDSEDVITSPA
Sbjct  601   TQSTRPPPPPDVGFSHPFSSDGEVGEGPIKPIKSELFKDKVSRGDVLIEQDSEDVITSPA  660

Query  661   RGIEGMRVMKRKRMMHEKPIKERSKRLRRVREGEENQDGKSLTSDETTSRRRQASRETLQ  720
             RGIEGMRVMKRKRMMHEKPIKERSKRLRRVREGEENQDGKSLTSDETTSRRRQASRETLQ
Sbjct  661   RGIEGMRVMKRKRMMHEKPIKERSKRLRRVREGEENQDGKSLTSDETTSRRRQASRETLQ  720

Query  721   AGYAALNKLLLRKLVDEALQAAAFPRTHPEFHTIWAWIVRGCAFSLRETFSTQELEETDL  780
             AGYAALNKLLLRKLVDEALQAAAFPRTHPEFHTIWAWIVRGCAFSLRETFSTQELEETDL
Sbjct  721   AGYAALNKLLLRKLVDEALQAAAFPRTHPEFHTIWAWIVRGCAFSLRETFSTQELEETDL  780

Query  781   MEPIQIHVQSYLPSSFRCSRSSSPLYTDVASPLPCDSSMLSQIESERLMENKAEDERWMQ  840
             MEPIQIHVQSYLPSSFRCSRSSSPLYTDVASPLPCDSSMLSQIESERLMENKAEDERWMQ
Sbjct  781   MEPIQIHVQSYLPSSFRCSRSSSPLYTDVASPLPCDSSMLSQIESERLMENKAEDERWMQ  840

Query  841   EGGLMALAEIWVAAGETREQKDRPVVSALAVGRKGERGDDSSDLSDEDSSSDSSSVLSPW  900
             EGGLMALAEIWVAAGETREQKDRPVVSALAVGRKGERGDDSSDLSDEDSSSDSSSVLSPW
Sbjct  841   EGGLMALAEIWVAAGETREQKDRPVVSALAVGRKGERGDDSSDLSDEDSSSDSSSVLSPW  900

Query  901   SSCPALCSTSSSNSDPAANPASSSLPDSESTSRDSTLRKRYPTFNPIRTHIPTLLTPHRA  960
             SSCPALCSTSSSNSDPAANPASSSLPDSESTSRDSTLRKRYPTFNPIRTHIPTLLTPHRA
Sbjct  901   SSCPALCSTSSSNSDPAANPASSSLPDSESTSRDSTLRKRYPTFNPIRTHIPTLLTPHRA  960

Query  961   VVSSRTRLSKPTSHVDPASTLDPRPDSNTAIHRELKNSHANPASDIDRRTPQQAVIDVVD  1020
             VVSSRTRLSKPTSHVDPASTLDPRPDSNTAIHRELKNSHANPASDIDRRTPQQAVIDVVD
Sbjct  961   VVSSRTRLSKPTSHVDPASTLDPRPDSNTAIHRELKNSHANPASDIDRRTPQQAVIDVVD  1020

Query  1021  EEETEDETWF  1030
             EEETEDETWF
Sbjct  1021  EEETEDETWF  1030


Lambda      K        H        a         alpha
   0.309    0.124    0.349    0.792     4.96 

Gapped
Lambda      K        H        a         alpha    sigma
   0.267   0.0410    0.140     1.90     42.6     43.6 

Effective search space used: 12644516594400


  Database: nr
    Posted date:  Sep 23, 2015 12:05 AM
  Number of letters in database: 26,053,659,533
  Number of sequences in database:  71,551,133


Matrix: BLOSUM62
Gap Penalties: Existence: 11, Extension: 1
Neighboring words threshold: 11
Window for multiple hits: 40
```
